# Cubeb (*Piper cubeba* L.): nutritional value, phytochemical profiling and dermacosmeceutical properties

**DOI:** 10.3389/fnut.2024.1352548

**Published:** 2024-05-21

**Authors:** Badr Eddine Drissi, Ismail Mahdi, Ahmet Buğra Ortaakarsu, Mohamed A. O. Abdelfattah, Widad Ben Bakrim, Sohaib Khatib, Mona F. Mahmoud, Latifa Bouissane, Mansour Sobeh

**Affiliations:** ^1^AgroBioSciences Program, College of Agriculture and Environmental Sciences, University Mohammed VI Polytechnic, Ben Guerir, Morocco; ^2^Molecular Chemistry, Materials and Catalysis Laboratory, Faculty of Sciences and Technologies, Sultan Moulay Slimane University, Beni-Mellal, Morocco; ^3^Department of Chemistry, Faculty of Science, Gazi University, Ankara, Turkey; ^4^College of Engineering and Technology, American University of the Middle East, Egaila, Kuwait; ^5^Department of Pharmacology and Toxicology, Faculty of Pharmacy, Zagazig University, Zagazig, Egypt

**Keywords:** *Piper cubeba*, dermacosmeceutical properties, nutritional contents, terpenoids, phytochemical profiling

## Abstract

**Introduction:**

Cubeb, *Piper cubeba* L., has been used for centuries in traditional medicine and culinary practices, with a wide range of biological and pharmacological activities.

**Objective:**

Herein, we determined the phytochemical profile, mineral, fatty acids, and amino acid contents of *P. cubeba* berries and assessed the dermacosmeceutical properties of their water extract and essential oil (EO). These included assessing their antioxidant and antibacterial activities as well as their *in vitro* inhibitory activities against tyrosinase and elastase enzymes. In addition, molecular docking and molecular dynamics studies were performed on the major identified compounds of the EO.

**Results and discussion:**

A total of forty-three compounds belonging to organic acids, phenolic acids and flavonoids were found in the water extract, while 36 volatile compounds were identified in the EO with Z-isoeugenol, dihydroeugenol, β-pinene, E-caryophyllene, and 1,8-cineole as major constituents. The berries were found to be rich in sodium and iron, have moderate zinc content along with low contents of total nitrogen, phosphorus, and potassium. Amino acid analysis revealed a considerable concentration of isoleucine and phenylalanine, whereas 11,14,17-eicosatrienoic acid and linoleic acid were identified as the major fatty acids. In the DPPH and FRAP assays, the water extract elicited considerable antioxidant activity compared to the reference compounds. Enzyme inhibitory assays revealed that the EO had a potential to inhibit tyrosinase and elastase enzymes with IC_50_ values of 340.56 and 86.04 μg/mL, respectively. The water extract and EO completely inhibited the bacterial growth at MIC of 50 mg/mL and 20%, respectively. At sub-MIC concentrations, the extract and the EO substantially reduced the biofilm formation by up to 26.63 and 77.77%, respectively, as well as the swimming and swarming motilities in a dose-dependent manner. Molecular docking and molecular dynamics showed that the five main components of *P. cubeba* EO could be the major contributors to the elastase and tyrosinase inhibitory effect.

**Conclusion:**

This study emphasizes the promising potential of *P. cubeba* as a valuable source of natural compounds that can be utilized for the development of innovative pharmaceuticals, dietary supplements, and dermacosmeceutical agents.

## Introduction

The skin is the largest organ in the human body that is in direct contact with the external environment. It regulates body temperature and water stress, hinders germs penetration, and provides substantial protection from UV radiation. The outermost layer of the skin, referred to as the stratum corneum, is a diverse and semi-permeable layer within the epidermis. It serves as a protective barrier against dehydration and retains an adequate amount of water necessary for its proper functioning. The increase in trans-epidermal water loss and improper skin hydration compromise the integrity of the stratum corneum and lead to an altered skin barrier, which have been found to be highly related to skin aging (1). Skin aging is mainly marked by the damage of the collagen and elastin fibers present in the dermal tissue of the skin. This leads to reduced skin elasticity, saggy skin, and wrinkles formation (2).

Antioxidants are well reported to decrease the rate of skin aging. The mechanisms of action involve the inhibition of the key enzymes associated with aging, specifically elastase and tyrosinase. Elastase is an essential enzyme responsible for breaking down major components of the skin’s extracellular matrix. On the other hand, tyrosinase is an enzyme involved in the conversion of tyrosine to melanin, the main contributor to skin pigmentation. Therefore, inhibiting elastase and tyrosinase activities plays a significant role in slowing down skin aging and owning a brighter complexion (3). Natural products and their derivatives have been used since ancient times as therapeutic and cosmetic agents. They are a rich source of antioxidants and other cosmo-therapeutics that can be used to delay skin aging and improve its health (4).

The genus *Piper* is a part of the Piperaceae family, which comprises over 700 species. *Piper* plants encompass a diverse range of forms, including erect and spreading herbs, shrubs, and occasional trees and possess considerable economic and medicinal importance. One notable species within this group is *P. cubeba*, which is native to Java and Borneo and commonly known as Java pepper in the local region. Significant populations of this species are commonly observed in Indonesia, India, North Africa, Southern and Western Europe (5). The plant is widely employed as a spice in traditional practices and mainly cultivated for its berries, which are known for their high content of essential oil (5). A diverse array of biological and pharmacological activities has been previously reported for *P. cubeba*. These include antioxidant, anti-cancer, anti-inflammatory, anti-diabetic, antiparasitic, antibacterial activities in addition to its ability to modulate melanogenesis (6–9). Furthermore, a recent study revealed promising hepato- and renoprotective activities by the plant (10–12).

The potent antioxidant, anti-inflammatory, and antimicrobial properties of the extracts and EO derived from *P. cubeba* make the plant’s ingredients highly suitable for application in the skincare and cosmetic industries (13). However, there has been very limited research conducted on the plant’s effects in dermatology and cosmetics.

In this work, we profiled the chemical composition of the water extract and the essential oil (EO) of cubeb berries using LC-ESI-MS and GC-MS, respectively. Moreover, the berries’ nutritional value with respect to mineral content, fatty acid and amino acid composition was determined. We also evaluated, *in vitro*, the dermacosmeceutical properties of both the water extract and EO by assessing the antioxidant and antibacterial activities in addition to the inhibitory activities against elastase and tyrosinase enzymes, implicated in skin senescence and aging. Finally, computational tools including molecular docking and molecular dynamics were employed to investigate the binding mode and the inhibitory potential of the oil’s major phytoconstituents against the two target enzymes.

## Materials and methods

### Chemicals and reagents

Acetonitrile (ACN, LCMS grade, Carlo Erba, Italie), water (LCMS grade, Merck, Germany), formic acid (FA, LCMS grade, Carlo Erba, Italie), 2,2-diphenyl-1-picrylhydrazyl (DPPH, 98%, Alfa Aesar, Germany), elastase from porcine pancreas (Sigma Aldrich, Germany), N-Succinyl-Ala-Ala-Ala-p-nitroanilide (Sigma Aldrich, Germany), tyrosinase from mushroom (Sigma Aldrich, Germany), 3,4-dihydroxy-L-phenylalanine (L-DOPA, Alfa Aesar, Germany), Mueller Hinton (MH, Biokar, France), and tris–HCl (Sigma Aldrich, Germany) were utilized in the current study. All other chemicals and reagents were of analytical grade.

### Plant material and extraction

*P. cubeba* berries were harvested at the Tafoughat Mountain in Berkane city (34°47′17.244′′; 2°21′42.776′′), Morocco (Voucher No: BD-112). The berries (20 g) were sorted, cleaned, grinded using RETSCH GM 200 and exhaustively extracted in ultrasonic machine (SONICS vibra cell, 1500-Watt, 20 kHz, time 20 min, Temp 5°C, Pulse 10 to 20 and Ampl 40%) with 200 mL of water to obtain a yield of 6.55% (w/w). Another portion (128 g) was extracted by hydrodistillation using a Clevenger type for 3 to obtain 1.9% of oil (v/w).

### High performance liquid chromatography (LC-ESI-MS/MS)

The LC-ESI-MS/MS was performed by Shimadzu Japan system, operated by LabSolutions software, and connected to MS 8050 mass spectrometer with an electrospray ionization source. A C18 reverse phase column (Zorbax Eclipse XDB-C18, fast resolution, 4.6 × 150 mm, 3.5 μm, Agilent, USA) was used for the separation process. The mobile phase and the mass spectrometry conditions were set according to the previously described method (14). Ions were detected in negative mode using a full scan mode within a mass range of 100–1500 *m/z* (Supplementary material).

### Gas chromatography (GC-MS)

Gas chromatography was performed by a SHIMADZU GCMS-TQ8040 coupled with a mass spectrometer (GC-MS) system. Separation was done by Rtx-5MS fused-bond column, provided by Restek, PA, USA, with 30 m length, 0.25 mm internal diameter, and 0.25 μm film thickness. The temperature program started at 50°C and increased at a rate of 5°C/min until reaching 300°C. A 3-min isotherm was maintained at 300°C. The injector temperature was set at 250°C, and helium was the carrier gas with a flow rate of 1.5 mL/min. For the mass spectrometer, the ion source temperature was set to 200°C, the interface temperature was set to 280°C, and the mass range was set to be from 50 to 500 *m/z*. The samples were diluted to a concentration of 1% v/v and injected in split mode. Compound identification was performed using the NIST 2017 database, and the retention indices (RIs) of the isolated components were calculated based on a set of standard n-alkanes that were analyzed under the same chromatographic conditions (Supplementary material).

### Mineral analysis and amino acids analysis

Total nitrogen was analyzed according to the Kjeldahl method (15), while other nutrient content of the plant was analyzed using ICP-AES (Agilent 5110, Santa Clara, California, USA). The dried biomass was ground separately and digested with 4 M HNO_3_ before being analyzed in the ICP. The Characterization and quantification of the amino acid were done using a SHIMADZU JAPAN system coupled to MS 8050 mass spectrometer according to (16) (Supplementary material).

### Fatty acids analysis

Fatty acid analysis was carried out according to the method described by (17) using GCMS (Supplementary material).

### Phytocontents and *in vitro* antioxidant activity

#### Total polyphenols content

The determination of total phenolic content (TPC) was carried out using Folin-Ciocalteu method according to (18). In brief, 20 μL of the extract were added to 100 μL of Folin–Ciocalteu reagent (1/10 dilution), incubated for 5 min, then 80 μL of 7.5% Na_2_CO_3_ (w/v) were added and mixed well. The reaction mixture was incubated for 30 min. The absorbance was measured spectrophotometrically at 765 nm and the TPC was expressed as milligrams of gallic acid equivalents (GAE) per gram of the extract.

#### Total flavonoids content

The quantification of flavonoids was based on the formation of a highly stable complex between aluminum chloride and the oxygen atoms at C-4 and C-5 of the flavonoid molecules according to (19). The reaction mixture consisted of 100 μL of extract, 50 μL AlCl_3_ (1.2%), and 50 μL of potassium acetate (120 mM). After allowing the mixture to stand for 30 min at room temperature, the absorbance was measured at 415 nm. The total flavonoid content (TFC) was expressed as milligrams of quercetin equivalent per gram of the extract.

#### Total sugar and protein contents

The total sugar content (TSC) was determined according to (20). In brief, 0.5 mL of the extract reacted with 4.5 mL of anthrone reagent (anthrone sulfuric acid) and the reaction mixture was then allowed to boil, in a water bath, for 7 min. After cooling, the absorbance was measured at 620 nm. The TSC was expressed as milligrams of glucose equivalent per gram of the extract. Total protein contents (TPrC) were determined using the Bradford assay (21) (Supplementary material). The assays were performed in triplicate.

### The 2,2-diphenyl-1-picrylhydrazyl (DPPH) and ferric reducing antioxidant power (FRAP) assays

The free radical DPPH scavenging assay was carried out following the previously described procedure by (22). The assay was performed in triplicate and the percentage reduction of the DPPH radical was calculated using the equation:


%DPPH=[A-controlA/sampleA]control×100]

The FRAP assay was carried out following the protocol modified by (23) for the microplate screening system. The assay was performed in triplicate and the antioxidant activity was expressed as mM FeSO_4_/mg extract (Supplementary material).

### Enzyme inhibitory activities

#### Elastase activity

Elastase activity was determined according to (24) with some minor modifications. In brief, 100 μL of 0.2 M tris-HCl buffer (pH 8.0) was mixed with 25 μL of 5 mM N-Succinyl-Ala-Ala-Ala-p-nitroanilide and 50 μL of the extract. The mixture was incubated at 25°C for 15 min. Then, 25 μL of elastase (0.3 units/mL) was added to the solution and the reaction mixture was incubated again at 25°C for 15 min. The absorbance was measured at 410 nm. Epigallocatechin gallate (EGCG) was used as a positive control and the percentage inhibition of elastase activity was calculated using the equation:


%elastaseinhibition=[A-controlA/sampleA]control×100].

#### Tyrosinase activity

The tyrosinase activity was determined using mushroom tyrosinase and L-DOPA as a substrate following the previously published protocol by (25) with some minor modifications. In brief, 88 μL of 2 mM L-DOPA, 0.1 M phosphate buffer (pH 6.5) and 10 μL of the extract were mixed and incubated at 25°C for 2 min. Subsequently, 20 μL of mushroom tyrosinase (200 units/mL) was added to the solution and the reaction mixture was incubated at 25°C for 10 min. After the incubation period, the absorbance was measured at 475 nm. Kojic acid was used as a positive control and the percentage inhibition of tyrosinase activity was calculated using the equation:


%tyrosinaseinhibition=[A-controlA/sampleA]control×100].

### Antibacterial activity

#### Determination of the minimum inhibitory concentration (MIC)

The broth microdilution assay was used to evaluate the effect of *P. cubeba* extract and EO on the growth of *Pseudomonas aeruginosa* (26). In brief, the highest concentration of either the extract or the EO was prepared in Mueller Hinton (MH) broth, filter-sterilized and sequentially diluted to prepare a series of concentrations varying from 100 to 6.25 mg/mL for the extract and from 20 to 0.625 % for the EO. Triplicates of each concentration were then transferred into the wells of a microplate. Afterward, the wells were inoculated with 2 μL of a fresh overnight culture of *P. aeruginosa* (OD_600nm_ = 1) and incubated at 37°C/150 rpm for 18 h. The lowest concentration that inhibited the visible microbial growth was regarded as MIC.

#### Biofilm inhibition assay

The antibiofilm potential of both the extract and EO against *P. aeruginosa* was performed using the crystal violet colorimetric assay (26, 27). The plant extract and EO at the doses of 1/8 and 1/4 MIC were used in this assay. Triplicates of each concentration were transferred into the wells of a microplate and inoculated with 2 μL of a fresh overnight culture of *P. aeruginosa* (OD_600nm_ = 1) and incubated at 37°C/150 rpm for 18 h. Bacteria-free media were used as negative controls. Following incubation, the wells were subjected to four washes using a PSB solution to remove free-floating bacteria. Then, 200 μL of a 1% crystal violet solution were added to the wells and vigorously washed with distilled water to eliminate the excess crystal violet. After drying for 15 min, the biofilm was solubilized by adding 200 μL of 95% ethanol and the absorbance was determined at OD_595nm_.

#### Swimming and swarming inhibition assays

To evaluate *P*. *cubeba* extract and EO effects on the motilities of *P. aeruginosa*, the swimming and swarming media (28) were supplemented with doses equivalent to 1/8 and 1/4 MIC of either the extract or EO. Next, 5 μL of *P. aeruginosa* (OD_600nm_ = 1) were inoculated at the center of each plate and incubated at 37°C for 24 h. The swimming and swarming diameters were measured in cm.

### Computational methods

#### Molecular docking

Virtual screening analyses, using molecular docking as computational tool, were conducted following the methodology described by (29). The major identified components from the essential oil of *P. cubeba* berries were subjected to virtual screening via molecular docking study using the MOE software (molecular operating environment), developed by Chemical Computing Group Inc., based in Montreal, QC, Canada. The docking targeted the binding active sites of two enzymes associated with aging, tyrosinase (PDB id: 2Y9X) and elastase (PDB id: 1Y93) and the structures of the enzymes were obtained from the Protein Data Bank (www.pdb.org). The docking protocol was validated by running self-docking of the co-crystallized ligand corresponding to each enzyme and the RMSD values were less than 2 Å.

#### Molecular dynamics simulation

The interface of Maestro Software (Schrödinger Release 2023-2, 2023) and its modules were used throughout the molecular dynamics’ simulation.

#### Protein preparation

Protein Preparation Wizard in Maestro was used to complete the missing amino acid residues in elastase and tyrosinase enzymes. The process was performed at pH 7.0, with the water molecules left undeleted. The hydrogen bonds between water molecules and the protein, ligand-protein, and ligand-water were optimized according to pH 7.0. Finally, an energy minimization process was performed using the OPLS3e force field (30, 31).

#### System setup

System Setup was performed using the different modules embedded in Maestro. In brief, the prepared protein structures were immersed in a solvent box with dimensions of 10 Å × 15 Å × 10 Å. The solvent molecules were modeled using Simple Point Charge (SPC) water model. Sodium ions were added to the medium to neutralize the system. Additionally, 0.15 M NaCl was added using the Monte Carlo method for modeling the physiological environment. Finally, the system setup process was started by selecting the force field OPLS3 (32, 33). Desmond module in Maestro package was used to perform the molecular dynamics simulation. For protein structures, the desired temperature, pressure, and particle NPT settings were selected, each as a constant throughout the simulation. The temperature was set to 300 K using a Nose-Hoover thermostat and the pressure was set to 1 bar using a Martyna-Tobias-Klein barostat. Molecular dynamics simulation took place over two stages; the relaxation stage (2 ps) and the simulation generation stage (100 ns) (34).

### Generalized born surface area (MM-GBSA) calculations

The MM-GBSA calculations provide insights about the stability of the ligand-protein complexes employed in the study. The calculations were monitored at 400 frame intervals throughout the entire molecular dynamics simulation, and the change in the energy of the formed complexes was analyzed as numerical data. For this purpose, the Prime module in Maestro package, employing VSGB solvent model and OPLS3 force field, was used (35).

### Statistical analysis

The obtained data was subjected to statistical analysis using one-way analysis of variance (ANOVA), followed by multiple comparisons using Dunnett’s test. The data was represented as the mean ± standard deviation from three independent experiments. Each treatment was compared to a control group. Statistical significance was defined at a *p*-value < 0.05.

## Results

### High performance liquid chromatography (LC-ESI-MS/MS)

**FIGURE 1:**
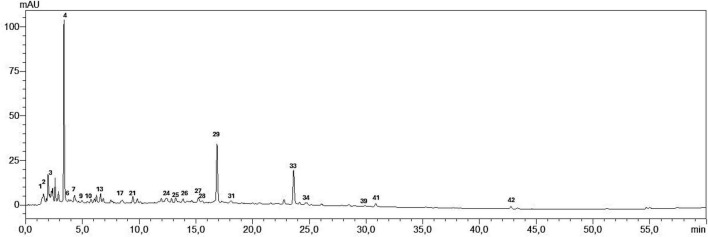
LC profile of *P. cubeba* berries water extract at 254 nm.

The identification of the compounds present in the water extract of *P. cubeba* was accomplished by utilizing their MS, MS^2^ fragments, and retention times. A comprehensive analysis revealed the identification of forty-three secondary metabolites within the extract, which encompassed organic acids, phenolic acids, and flavonoids (Figure 1 and Table 1).

**TABLE 1:** Composition of the water extract from *P. cubeba* berries using LC-MS/MS analysis.

Peak no.	R_*t*_ (min)	[M-H]^–^	MS/MS	Annotated metabolites
1	1.52	191	111	Citric acid^a^
2	1.69	133	115	Malic acid^a^
3	2.23	331	169	Galloyl hexose
4	3.25	609	305, 441	(epi)Gallocatechin digallate^c^
5	3.31	169	125	Gallic acid^b^
6	3.5	483	169, 331	Digalloyl hexose
7	4.28	609	305, 441	(epi)Gallocatechin digallate isomer^c^
8	4.88	315	153	Dihydroxybenzoic acid hexoside^b^
9	5.00	483	169, 331	Digalloyl hexose
10	5.79	341	179	Caffeoyl hexose^b^
11	6.02	153	109	Dihydroxybenzoic acid
12	6.09	593	305	(epi)Gallocatechin-(epi)catechin^c^
13	6.50	593	289	(epi)Catechin-(epi)gallocatechin^c^
14	6.51	299	121	Benzoic acid derivative
15	7.04	483	169, 331	Digalloyl hexose
16	8.12	285	153	Dihydroxybenzoic acid pentoside^b^
17	8.24	399	181	Unknown
18	9.03	329	167	Vanillic acid hexoside^b^
19	9.09	137	108	Hydroxybenzoic acid^b^
20	9.27	633	301	Galloyl-Hexahyroxydiphenoyl(HHDP)-hexose^d^
21	9.33	577	289	(epi)-Catechin-(epi)-catechin^c^
22	10.77	295	179	Caffeic acid malate^b^
23	12.16	633	301	Galloyl-HHDP hexose^d^
24	12.4	305	179	(epi)Gallocatechin^c^
25	13.17	935	301	Di-HHDP-galloyl-hexose^d^
26	14.02	163	119	Coumaric acid^b^
27	15.39	609	301	HHDP derivative
28	15.47	467	169	Gallic acid coumaroyl gallate^b^
29	16.78	477	301	HHDP derivative
30	17.02	511	169	Galloyl hexose acetyl derivative
31	17.99	463	301	HHDP derivative
32	20.21	493	317	Myricetin glucuronide^e^
33	23.46	463	301	Quercetin hexoside^e^
34	24.60	477	301	Quercetin glucuronide^e^
35	25.80	483	169, 331	Digalloyl hexose
36	25.99	615	301	Quercetin galloyl hexose^e^
37	27.00	447	285	kaempferol hexoside^e^
38	28.33	447	301	Quercetin deoxyhexoside^e^
39	29.83	599	285	Kaempferol galloyl hexoside^e^
40	30.13	383	170	R-Methylcubebin
41	31.01	493	169	Caffeoyl galloyl hexose^b^
42	42.52	467	331, 169	Dihydroxy benzoyl galloyl hexose^b^
43	53.57	331	169	Gallic acid derivative

a: organic acid, b: phenolic acid, c: condensed tannins, d: hydrosable tannins, e: flavonoids.

### Gas chromatography (GC-MS)

**FIGURE 2:**
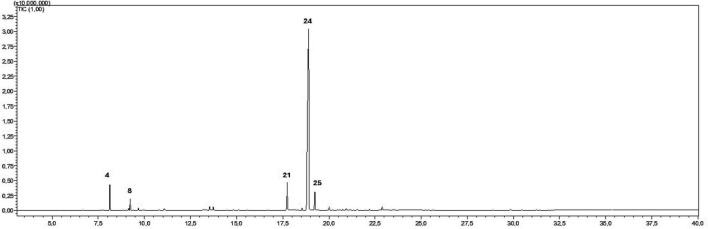
GC profile of *P. cubeba* berries essential oil.

Analysis of *P. cubeba* essential oil revealed the presence of thirty-six compounds (Table 2). The major compounds were Z-isoeugenol with a percentage of 76.99% followed by dihydroeugenol with 5.76% and β-pinene, E-caryophyllene, 1,8-cineole (4.21, 3.67, and 2.01%, respectively) (Figure 2). The remaining compounds were present in the EO at concentrations lower than 1%.

**TABLE 2:** Essential oil composition of *P. cubeba* berries using GC-MS analysis.

Peak N°	Compounds	Relative abundance (%)	RI calc	RI Adams
1	Tricyclene	0.04	911	921
2	α-Pinene	0.05	953	932
3	Sabinene	0.06	956	969
4	β-Pinene	4.21	971	974
5	δ-2-Carene	0.07	1000	1001
6	δ-3-Carene	0.09	1008	1008
7	Limonene	0.38	1013	1024
8	1,8-Cineole	2.01	1016	1026
9	Z-β-Ocimene	0.36	1034	1032
10	δ-Terpinene	0.11	1047	1054
11	Terpinolene	0.14	1080	1086
12	Linalool	0.39	1092	1095
13	Terpinen-4-ol	0.32	1178	1174
14	α-Terpineol	0.10	1184	1186
15	Methyl chavicol	0.84	1192	1195
16	γ-Terpineol	0.59	1200	1199
17	*cis*-Carveol	0.02	1230	1226
18	Cuminal	0.08	1244	1238
19	Chavicol	0.03	1256	1247
20	2,4-Decadienal	0.06	1321	1318
21	Dihydroeugenol	5.76	1361	1366
22	α-Copaene	0.12	1378	1374
23	β-Elemene	0.45	1393	1389
24	*Z*-Isoeugenol	76.99	1408	1406
25	*E*-Caryophyllene	3.67	1421	1417
26	α-Humulene	0.68	1452	1452
27	γ-Gurjunene	0.07	1471	1475
28	Germacrene D	0.18	1477	1480
29	β-Selinene	0.20	1482	1489
30	E-Methyl isoeugenol	0.48	1489	1491
31	E,E-α-Farnesene	0.04	1496	1505
32	δ-Amorphene	0.02	1507	1511
33	δ-Cadinene	0.13	1517	1522
34	Elemicin	0.16	1552	1555
35	Caryophyllene oxide	0.84	1588	1582
36	Neointermedeol	0.25	1665	1658
Monoterpene hydrocarbon		5.51		
Oxygenated monoterpenes		3.43		
Sesquiterpene hydrocarbons		5.56		
Oxygenated sesquiterpene		0.84		
Phenylpropanoids		84.26		
Others		0.14		
Total components		99.74		

### Nutritional value

*P. Cubeba* berries showed to contain high amounts of sodium and iron, moderate amounts of zinc, and relatively low amounts of total nitrogen, phosphorus and potassium, magnesium, and calcium (Table 3). Isoleucine and phenylalanine were present in considerable concentrations (563.76 and 334.71 mg/kg, respectively) compared to the other amino acids (Table 4). Additionally, the analysis of fatty acids revealed that 11,14,17-eicosatrienoic acid and linoleic acid were the predominant members (58.66 and 17.42%, respectively) (Table 5).

**TABLE 3:** Mineral composition of *P. cubeba* berries.

Parameter	Unit	Results	Method used
Total Nitrogen	%	1.14	Kjeldahl
Total Phosphorus	%	0.10	ICP-OES
Total Potassium	%	1.50	
Magnesium	%	0.13	
Calcium	%	0.71	
Sodium	mg/kg	161.67	
Iron	mg/kg	64.75	
Zinc	mg/kg	4.69	

**TABLE 4:** Amino acid composition of *P. cubeba* berries.

Compounds	Concentration (mg/kg)
Aspartic acid	11.62
4-Hydroxyproline	22.39
Glutamic acid	19.66
Threonine	12.30
Glutamine	16.95
Alanine	13.98
Valin	36.35
Lysin	43.16
Histidine	32.60
Arginine	116.19
Tyrosine	46.23
Isoleucine	563.76
Phenylalanine	334.71
Tryptophan	4.40

**TABLE 5:** Fatty acids composition of *P. cubeba* berries.

Compounds	Relative abundance (%)
Palmitic acid^a^	5.74
Linoleic acid^a^	17.42
11,14,17-Eicosatrienoic acid^a^	58.66
Stearic acid^a^	5.18
Linoleyl acetate	5.24
Oleic acid derivative	2.76
Myristic acid derivative	5.00

^a^The compounds were identified as methyl ester.

### Dermacosmeceutical properties

#### *In Vitro* antioxidant activity

Compared to *P. cubeba* EO that showed a modest antioxidant activity, the plant’s water extract showed powerful antioxidant potential in DPPH and FRAP assays. The substantial antioxidant activity of the extract could be attributed to the high content of polyphenols and flavonoids that are not present in the EO as shown in Table 6.

**TABLE 6:** *In vitro* antioxidant activity of the water extract and essential oil of *P. cubeba*.

Sample	Water extract	Essential oil	Ascorbic acid	Quercetin
TPC (mg GAE /g Extract)	132.39 ± 9.87	_	_	_
TFC (mg QUE/g Extract)	16.49 ± 3.14	_	_	_
TSC (mg Glu/g Extract)	93.18 ± 2.11	_	_	_
TPrC (mg BSA/g extract)	192.68 ± 3.90	_	_	_
DPPH IC_50_ (μg/mL)	54.02 ± 7.22^b^	3448.33 ± 84.62^a^	3.89 ± 0.65	3.93 ± 0.73^c^
FRAP (mM FeSO_4_/mg extract)	27.71 ± 4.98^a^	0.364 ± 0.01^b^	31.48 ± 7.58^a^	34.15 ± 8.8^a^

Letters in superscript (a, b, and c) indicate a statistical difference at *p* < 0.05. TPC, Total Polyphenols Content; TFC, Total Flavonoids Content; TSC, Total Sugar Content; TPrC, Total Proteins Content; DPPH, 2,2-diphenyl-1-picrylhydrazyl; FRAP, Ferric Reducing Antioxidant Power.

#### Enzymes inhibitory activities

The water extract and EO of *P. cubeba* were investigated for their *in vitro* inhibitory activity against elastase and tyrosinase enzymes, associated with skin aging. It was noticed that the EO was a more powerful inhibitor against the two enzymes than the water extract, where the IC_50_ values of the oil were much lower than those of the water extract. In general, both oil and the extract showed moderate inhibitory activity against elastase and tyrosinase enzymes when compared to kojic acid and EGCG, the reference inhibitors (Table 7).

**TABLE 7:** *In vitro* inhibitory activities of *P. cubeba* berries water extract and essential oil (EO) against elastase and tyrosinase enzymes.

		IC_50_ (μg/mL)	
Sample	Extract	Tyrosinase	Elastase
Berries	Water extract	259.6 ± 27.03^b^	180,23 ± 9,02^b^
	Essential oil (EO)	340.56 ± 11.42^a^	86.04 ± 13.41^c^
Reference	Kojic acid	24.18 ± 3.41^c^	–
	EGCG	–	231.53 ± 7.08^a^

EGCG, Epigallocatechin gallate. Letters in superscript (a, b, and c) indicate a statistical difference at *p* < 0.05.

#### Antibacterial activity

**FIGURE 3:**
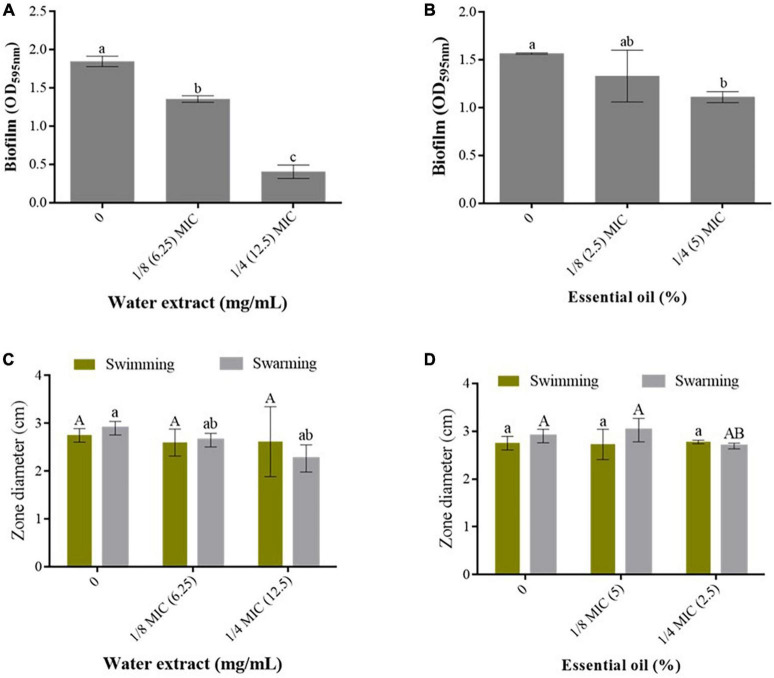
Effect of *P. cubeba* berries water extract and essential oil on *P. aeruginosa* biofilm production **(A,B)** and swimming and swarming of *P. aeruginosa* grown in absence and presence of the extract or the essential oil at the sub-MICs **(C,D)**.

In this study, *P. cubeba* berries water extract and EO exhibited complete inhibition of *P. aeruginosa*, at MIC values of 50 mg/mL and 20%, respectively. At subinhibitory concentrations of 1/8 MIC (12.5 mg/mL) and 1/4 MIC (6.25 mg/mL), the water extract significantly reduced biofilm production by 26.63% and 77.77%, respectively (Figure 3A). Similarly, at 1/4 MIC (5%), the EO led to a significant decrease in biofilm production by 29.48% (Figure 3B). In addition, *P. cubeba* water extract demonstrated a dose-dependent reduction in *P. aeruginosa* swimming and swarming motilities, with reductions of up to 5.45 and 22.06%, respectively. However, no significant changes were observed compared to the extract-free media (Figure 3C). Likewise, the EO at 1/4 MIC (5%) %) resulted in a non-significant decrease in *P. aeruginosa* swarming motility, reaching up to 7.24%, while no significant effects were observed on bacterial swimming ability (Figure 3D).

### Computational findings

#### Molecular docking

A virtual molecular docking of the five major oil’s components was conducted to gain some insights about the components that are more likely responsible for the oil’s inhibitory potential against the two enzymes. Moreover, this kind of *in silico* study would elaborate as well on the molecular interactions between the oil’s components and the amino acid residues at the binding sites of the target enzymes. The docking score values and the molecular interactions between the studied enzymes and the five major components identified in *P. cubeb* essential oil are presented in (Table 8).

**TABLE 8:** Score functions and interactions of the major components identified in *P. Cubeba* essential oil upon docking to Tyrosinase and Elastase active sites.

	Tyrosinase (2Y9X)		Elastase (1Y93)	
Compounds	Score (kcal/mol)	Interactions	Score (kcal/mol)	Interactions
β-pinene	−7.53	His263 (hydrophobic interaction)	_	_
1,8-cineole	−7.11	His263 (hydrophobic interaction)	−10.10	Ala184 (hydrogen bond through water)
Dihydroeugenol	−9.78	His61 (hydrogen bond), His 263 (hydrophobic interaction), Val283 (hydrophobic interaction)	−11.90	Leu181 (hydrogen bond through water)
Z-isoeugenol	−10.15	His61 (hydrogen bond), His 263 (hydrophobic interaction), Val283 (hydrophobic interaction)	−10.32	Leu181 (hydrogen bond through water)
E-caryophyllene	−8.48	His85 (hydrophobic interaction)	_	_
Kojic acid (reference inhibitor)	−8.14	Cu^2+^ chelation, His 263 (hydrophobic interaction), Val283 (hydrophobic interaction)	_	
Epigallocatechin gallate (reference inhibitor)	_	_	−20.06	His57 (hydrogen bond).

**FIGURE 4:**
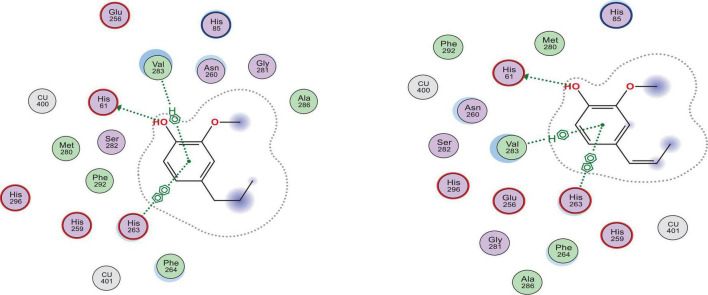
2D docking poses of dihydroeugenol **(left)** and Z-isoeugenol **(right)** upon docking into the binding site of tyrosinase enzyme.

The five docked compounds fitted properly in the binding site of tyrosinase enzyme, indicated by their docking score values that ranged from −10.15 to −7.11 kcal/mol and the molecular interactions they afforded with the amino acid residues at the target protein’s binding site (Table 8). Z-isoeugenol and dihydroeugenol showed the best binding affinity toward tyrosinase as they had docking scores of −10.15 and −9.78 kcal/mol, respectively, compared to the reference inhibitor, kojic acid (−8.14 kcal/mol). The two compounds afforded one hydrogen bond interaction with His61 and two hydrophobic interactions with His263 and Val283 residues (Figure 4).

**FIGURE 5:**
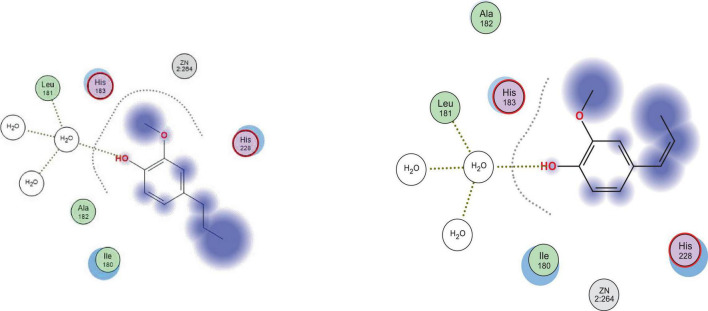
2D docking poses of dihydroeugenol **(left)** and Z-isoeugenol **(right)** upon docking into the binding site of elastase enzyme.

Regarding elastase enzyme, only dihydroeugenol, Z-isoeugenol, and 1.8-cineole were able to fit in and afford a hydrogen bond interaction with either Leu81 or Ala184 at the binding site of the enzyme. Similarly, the two terpenoids, dihydroeugenol and Z-isoeugenol, among the other oil components, showed the best binding affinity to elastase as they had the lowest minimum docking scores of −11.90 and −10.32 kcal/mol, respectively, compared to the reference inhibitor, epigallocatechin gallate (−20.06 kcal/mol). Both compounds afforded hydrogen bond interaction with Leu181 through some water molecules (Figure 5).

#### Molecular dynamics

Molecular docking showed that Z-isoeugenol and dihydroeugenol had the best binding affinities toward elastase and tyrosinase enzymes, thus they were employed in a molecular dynamics simulation to evaluate the stability of their ligand-protein complexes and confirm their binding affinity toward the two enzymes.

### Trajectory analysis

Trajectory analysis, including visual and animated versions of molecular dynamics simulations, showed that Z-isoeugenol and dihydroeugenol, bound to the elastase enzyme, were separated from the protein structure immediately after the simulation started and never found in the region where they first bonded. The elastase enzyme was therefore not included in further analyses.

The situation was different with the tyrosinase enzyme as the two ligands started the simulation by coordinating with the cupper atoms in the enzyme’s binding site and did not separate from the protein structure. Since the ligands had small molecular volume, they almost did not change conformation. The bond formed by the ligands with the cupper atoms was always preserved and there was no notable change in bond lengths.

In the Z-isoeugenol-tyrosinase complex, the two copper atoms interacted with the oxygen atom of the ligand’s hydroxyl group. This interaction was preserved throughout the whole simulation. The ligand sometimes exhibited some limited oscillatory movements around this coordination with the metal atom, however, the coordination was simultaneously restored, and the complex structure created a plateau level.

Similarly, the dihydroeugenol-tyrosinase complex showed the same binding modes as with Z-isoeugenol. The difference between the two complex structures was due to the differentiation of the aliphatic group in the part of the molecule that coordinated with the copper atoms and did not participate in the interactions associated with the hydroxyl group.

### Root-mean-square deviation (RMSD) of atomic positions

**FIGURE 6:**
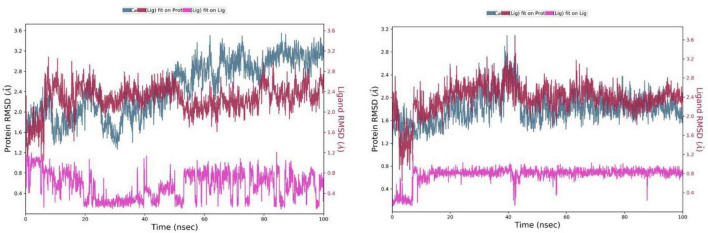
RMSD values of dihydroeugenol **(left)** and Z-isoeugenol **(right)** complexes with tyrosinase enzyme over a period of 100 ns. The red graph shows the deviation of the alpha-carbons of the protein structure relative to the ligand position. The gray graph represents the deviation of the alpha-carbons of the protein from the initial frame of the simulation and the pink graph represents the average deviation of the ligand from its initial position.

Root-mean-square deviation plots provide useful insights about the variation of the average positions of the ligand and the protein from their original conformation or relative to each other over the simulation time. The average RMSD values of both dihydroeugenol and Z-isoeugenol were 0.8 Å. The RMSD plot of dihydroeugenol-tyrosinase complex showed a rising trend from 2 Å to 3.2 Å and the ligand was bound with a restricted movement of the protein, then the average position of the protein structure decreased to around 2.4 Å and formed a plateau level in this region. The RMSD values of the Z-isoeugenol-tyrosinase complex shaped similar data (Figure 6). The low RMSD values of both ligands and the early plateau level attained by the ligand-protein complexes at around 10 ns of the simulation time indicate the complexes’ stability and confirm that the ligands remained bound to the tyrosinase binding site throughout the simulation time.

### Root mean square fluctuation (RMSF) of atomic positions

**FIGURE 7:**
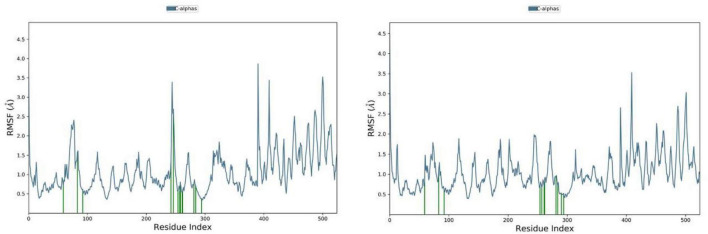
RMSF plots of dihydroeugenol **(left)** and Z-isoeugenol **(right)** complexes with tyrosinase enzyme over a period of 100 ns. Green vertical lines indicate the residues contacted by the ligands.

RMSF plots are useful for detecting local changes and fluctuations in the protein structure under investigation. The local fluctuations in the dihydroeugenol-tyrosinase complex were higher than in the Z-isoeugenol-tyrosinase complex (Figure 7). However, the contact points to the amino acid residues below 100 were almost the same in the two complexes. The fact that Z-isoeugenol caused fewer fluctuations might be related to the few differences in the contacts with the amino acid residues in the region between 200 and 300, which could have damped the fluctuations in the Z-isoeugenol-tyrosinase complex. In general, the fluctuations in the complexes formed by dihydroeugenol and Z-isoeugenol were as low as 0.5 and 0.1 Å, respectively, which confirmed the stability of the ligands in the protein cavity.

### Generalized born surface area (MM-GBSA)

**FIGURE 8:**
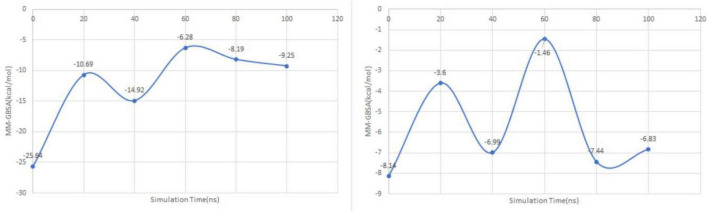
MM-GBSA plots of dihydroeugenol **(left)** and Z-isoeugenol **(right)** complexes with tyrosinase enzyme over a period of 100 ns.

To analyze the variation of the complexes’ stability throughout the molecular dynamics simulation, the potential free energies were successfully analyzed by MM-GBSA calculation (Figure 8).

The variation of MM-GBSA values showed that dihydroeugenol-tyrosinase complex reached a plateau level in the last 40 ns of the simulation, while Z-isoeugenol-tyrosinase complex showed a plateau level with MM-GBSA values fluctuating in a narrow range from the beginning of the simulation. The free binding energy of the dihydroeugenol-tyrosinase at the end of the simulation was −9.25 kcal/mol relative to −6.83 kcal/mol for the Z-isoeugenol, which indicates higher stability of the former complex.

## Discussion

*Piper cubeba* is a species of the Piperaceae family that is commonly used traditionally as food spices. Studies on this species have garnered attention because of its wide range of health benefits (5). However, further examination of the existing literature revealed that the nutritional value, LC-MS analysis of water extracts from the plant’s berries and its dermacosmeceutical properties have not been previously investigated. The current study identified the phytochemical composition of the water extract and the essential oil of the plant’s berries and determined its nutritional value with respect to mineral and amino acids contents. The study investigated as well the dermacosmeceutical properties of the plant’s berries by evaluating, *in vitro*, the antioxidant, anti-tyrosinase, anti-elastase and antibacterial activities of its water extract and EO. Computational tools including molecular docking and molecular dynamics simulations were employed to investigate the potential of the major components of the EO to bind to tyrosinase and elastase enzymes associated with skin aging and senescence. These *in silico* studies helped also to evaluate the ligands’ binding modes and the stability of the formed ligand-protein complexes to pinpoint the compounds that are most likely responsible for the inhibitory effect against the two enzymes.

The LC-MS/MS analysis led to the tentative identification of forty-three phytoconstituents from *P. cubeba* berries water extract. The predominating compounds belonged to phenolic acids (e.g., gallic acid, coumaric acid, galloyl glucose, and caffeoyl glucose), proanthocyanidins (e.g., (epi)gallocatechin, (epi)catechin-(epi)gallocatechin, and (epi)-catechin-(epi)-catechin), ellagitannins (e.g., hexahydroxydiphenoyl-D-glucose), and flavonoids (e.g., myricetin glucuronide, quercetin glucoside, and kaempferol glucoside). Analysis of the berries’ essential oil revealed thirty-six constituents with β-pinene, Z-isoeugenol, dihydroeugenol, 1,8-cineole and E-caryophyllene as the major ones. According to previous studies, *P. cubeba* EO was shown to contain eugenol, cubebene, caryophyllene, and pinene as the main compounds (36, 37). The percentage and composition of volatile oils can vary due to various intrinsic and extrinsic factors. These include the geographical origin of the plant, its phenological stage, the specific plant part used, harvesting period, methods of drying, storage conditions, and extraction techniques. These factors collectively influence the nature and proportions of volatile compounds present in the plant (38).

While the mineral content of medicinal plants can vary significantly based on various intrinsic and extrinsic factors, general trends have indicated that *P. cubeba* typically exhibits high levels of sodium and iron, moderate levels of zinc and potassium, and relatively low levels of total phosphorus and magnesium. This comes in accordance with our findings regarding the mineral composition of berries (Table 4). Compared to other *Piper* species, a study by (39) reported that both the leaves and seeds of *P. guineense* contained low mineral concentrations. Apart from the mineral content, we conducted amino acid analysis, which showed that *P. cubeba* berries were rich in isoleucine and phenylalanine with the concentration of 563.76 and 334.71 mg/kg, respectively, while moderate concentration of arginine was detected (116.19 mg/Kg). The analysis of fatty acids in *P. cubeba* revealed that 11,14,17-eicosatrienoic acid dominated at 58.66%, with linoleic acid following closely at 17.42%. Additionally, palmitic acid, stearic acid, and linoleyl acetate are present in moderate proportions (Table 5). In a separate investigation conducted by (9), the dichloromethane fraction exhibited the presence of dodecanoic acid at 24.05%, hexadecanoic acid at 11.37%, and 9-octadecenoic acid at 10%.

There has been a surge recently in the demand for skin care and antiaging dermal products as the world population is aging up. In this regard, cosmeceuticals stand as a new class of products that come between pharmaceuticals and cosmetics that aim to enhance the health and the beauty of the skin (40). While there is extensive literature supporting the traditional use of *P. cubeba* in medicine, there is a lack of studies focusing, specifically on its dermatological applications. Therefore, in this study, the dermacosmeceutical properties of *P. cubeba* berries were investigated with respect to antioxidant, antibacterial, anti-elastase, and anti-tyrosinase activities, which is a novel and unique aspect to be addressed in this work.

The water extract of the berries displayed moderate antioxidant potential in DPPH and substantial in FRAP assays as it showed comparable IC_50_ values to the reference drugs, ascorbic acid, and quercetin (Table 6). The extract exhibited potent antioxidant capacity in trapping DPPH free radicals, measured at a value of 54.02 ± 7.22 μg/mL, whereas ascorbic acid and quercetin demonstrated values of 3.89 ± 0.65 and 3.93 ± 0.73 μg/mL, respectively. Furthermore, the aqueous extract displayed significantly high reducing power activity compared to the references, with a value of 27.71 ± 4.98 mM FeSO_4_/mg extract, while ascorbic acid and quercetin exhibited values of 31.48 ± 7.58 mM FeSO_4_/mg extract and 34.15 ± 8.8 mM FeSO_4_/mg extract, respectively. Comparing the essential oil to the extract and the references, it’s evident that the former exhibits markedly lower antioxidant activity than the latter. This could be attributed to the richness of the water extract with considerable amounts of phenolics (132.39 mg GAE /g extract), and flavonoids (16.49 mg QUE/g extract) that are renowned with their powerful antioxidant activities. Noteworthy, the antioxidant activity of the ultrasound-assisted water extract of *P. cubeba* in this study surpassed that of the previously reported methanol, hexane, dichloromethane and hydroalcoholic extracts (58.75 μg/mL, 10 mg/mL, 650 μg/mL, 500 μg/mL, respectively) (7, 41, 42). The EO, on the contrary, displayed low antioxidant activity relative to the water extract and the reference drugs. However, another study showed that the plant’s EO from Saudi Arabia exhibited modest antioxidant activity at concentrations of 110 μg/mL in DPPH and 106 μg/mL in FRAP assays, which could be due to different composition than our EO from Morocco (43).

Elastase and tyrosinase are metalloprotease enzymes that are greatly involved in skin aging process and their overexpression during wound healing might impair the healing process leading to a chronic wound and other skin complications (44). The EO of *P. cubeba* berries showed appreciable *in vitro* inhibitory activity against elastase and tyrosinase enzymes (Table 7). We employed molecular docking and molecular dynamics simulations computational tools to investigate the binding mode and affinity of the essential oil’s major components toward elastase and tyrosinase enzymes. Molecular docking revealed that dihydroeugenol and Z-isoeugenol had the best binding affinity (lowest minimum scoring function) toward the two target enzymes. The most crucial structural feature of a ligand to bind a metalloprotease is its ability to coordinate with the metal atoms in the binding site. As shown in our MD study, the two compounds could not afford Zn^2+^ coordination at elastase binding site, detached off the enzyme at the very beginning of the simulation and scattered into the solvent environment. Despite that dihydroeugenol and Z-isoeugenol did not show metal coordination in the molecular docking, the MD results showed the potential of the two ligands to coordinate Cu^2+^ ions at the binding site of tyrosinase and to remain attached to the enzyme toward the end of the simulation. Comparing the binding modes of both ligands to tyrosinase and elastase enzymes, they afforded more hydrogen bonds and other non-covalent interactions with tyrosinase enzyme and preserved these interactions throughout the whole simulation time. Our *in-silico* findings pointed out that Z-isoeugenol and dihydroeugenol are most probably the main contributors to the enzyme inhibitory effects of the EO. Therefore, these two compounds could be potential hits for developing novel metalloproteases inhibitors of natural origin. Further clinical investigations are required to affirm the compounds’ efficiency to interfere with the target enzymes.

In dermatology, skin infections, especially of wounded skin, pose a significant threat to human health and can result in prolonged hospital stays, increased healthcare costs, and even mortality. Nosocomial infections are important hospital-acquired infections that still need efficient control strategies and treatment. Among the responsible agents, *Pseudomonas aeruginosa* is a major opportunistic bacterium that causes up to 57% of the occurring nosocomial infections (45). Within *P. aeruginosa* infections, cutaneous manifestations are one of the most important symptoms (46). When *P. aeruginosa* infects wounds, it often produces distinct clinical features such as necrotic skin lesions that may progress to tissue destruction. Several plant species have been recognized for their ability to combat bacterial infections and reduce microbial pathogenicity by targeting some significant virulence factors such as the bacterial biofilm and swimming and swarming motilities (47, 48). These two factors play a crucial role in the virulence of bacteria, enabling them to establish secure bacterial communities and traverse biological surfaces effectively (49, 50). Herein we showed that the *P. cubeba* berries water extract and EO completely inhibited the growth of *P. aeruginosa* at MIC of 50 mg/mL and 20%, respectively. Moreover, the water extract at 1/4 sub-MIC significantly decreased the bacterial biofilm production while both sub-MICs of the EO significantly counteracted the formation of the biofilm. In addition, the extract reduced the swimming and swarming of the bacterium in a dose dependent manner. The EO as well showed moderate effects on the bacterial swimming and swarming at the highest concentration tested. Comparatively, a study by (51) reported that the organic extracts (ethanol, acetone, and methanol) from *P. cubeba* fruits exhibited varying degrees of antibacterial activity against several bacterial strains, including *Staphylococcus aureus*, *Klebsiella* sp., *Escherichia coli*, *Enterobacter* sp., *Enterococcus* sp., and *P. aeruginosa*. The most potencies were obtained against *Enterococcus* sp. followed by *E. coli* and *P. aeruginosa*. Interestingly, *P. cubeba* EO exhibited potent antibacterial effects, particularly against *Listeria monocytogenes* and *S. aureus*. When applied as a topical cream, the oil expedited the wound healing process, elevated the superoxide dismutase level, and decreased the concentration of malondialdehyde (36). Moreover, the oil was reported to damage the microbial cytoplasmic membrane and the cell wall proteins involved in forming the biofilm in methicillin-resistant *S. aureus* (52). Here, we showed, for the first time, that *P. cubeba* berries water extract and EO components are potent in weakening *P. aeruginosa* virulence through reducing its capacity to form biofilm . This effect on biofilm production could be attributed to the ability of *P. cubeba* phytoconstituents to trigger bacterial genes involved in biofilm production such as pslA, pelA, and ppyR (53). These findings propose *P. cubeba* as an intriguing candidate for further exploration in the field of cosmeceutical agents.

## Conclusion

In the present study, we showed the remarkable nutritional properties, dermatological and antibacterial activities of *P. cubeba* berries. The plant was found to harbor high concentrations of sodium, iron, isoleucine, phenylalanine, 11,14,17-eicosatrienoic acid and linoleic acid. The water extract of the plant’s berries exhibited stronger antioxidant and anti-tyrosinase activities than the essential oil, yet the latter exhibited better inhibitory potential against elastase enzyme. Regarding the plant’s antibacterial potential, both water extract and EO inhibited the growth of *P. aeruginosa*, reduced biofilm formation and slightly hindered its swimming and swarming motilities. Computational simulations showed that the major EO components, especially Z-isoeugenol and dihydroeugenol, had good binding affinities and afforded significant interaction at the binding sites of elastase and tyrosinase enzymes. Nevertheless, additional investigations are required to delve into the complete capabilities of *P. cubeba* using cell-based assays and animal models, explore its modes of action and potential skin toxicities, and develop efficient formulations for different skin disorders.

## Data availability statement

The raw data supporting the conclusions of this article will be made available by the authors, without undue reservation.

## Author contributions

BD: Writing – original draft, Methodology, Investigation, Conceptualization. IM: Writing – original draft, Investigation. AO: Writing – original draft, Investigation. MA: Writing – original draft, Investigation. WB: Writing – original draft, Investigation. SK: Writing – original draft, Investigation. MM: Writing – review and editing, Conceptualization. LB: Writing – review and editing, Conceptualization. MS: Writing – review and editing, Supervision, Methodology, Investigation, Conceptualization.
