# Supplementary material for: Cubeb (Piper cubeba L.): nutritional value, phytochemical profiling and dermacosmeceutical properties
Source: Front Nutr. 2024 May 21;11:1352548. doi: 10.3389/fnut.2024.1352548 (PMC11148373; doi:10.3389/fnut.2024.1352548)
Supplement: Supplementary file 1 [file Data_Sheet_1.pdf]

Supplementary material

High Performance Liquid Chromatography

Following the extraction and freeze-drying process, 5 mg of the extract was dissolved in 1 ml of LCMS-grade water. The resulting solution was then filtered through a 0.22 µm syringe filter into vials. Finally, 5 µL volume of the filtered solution was injected into the machine Shimadzu Japan system connected to MS 8050 mass spectrometer using an autosampler SIL-40xs, Shimadzu. Ions were detected in negative mode using a full scan mode within a mass range of 100-1500 m/z, the ionization is carried out by ESI, Ag was used as the carrier gas for fragmentation and collision energy at 35 V.

Gas Chromatography

Following the extraction of essential oil, the sample was diluted to a concentration of 1% v/v with hexane. The resulting solution was then filtered through a 0.22 µm syringe filter into vials. Finally, 1 µL volume of the filtered solution was injected into the machine SHIMADZU GCMS-TQ8040 coupled with a mass spectrometer system using an autosampler in split mode HTA (HT2800T) (Brescia, Italy). The mass spectrometer, the ion source temperature was set to 200°C, the interface temperature was set to 280°C, and the mass range was set to be from 50 to 500 m/z.

Analysis of Amino Acids

Amino acid characterization and quantification were analyzed using a Shimadzu Japan system coupled to an MS 8050 mass spectrometer. Forty mg of the plant extract was hydrolyzed in 10 mL of 6 M HCl for 22 h at 110°C.The hydrolyzed sample was cooled down to 4°C to stop the hydrolyzation process and then diluted in 50 mL of distilled water. The hydrolysate pH was adjusted to 4.5 and filtered using a 0.22-micrometer PTFE membrane to remove suspended particles. Liquid chromatography was performed at 40°C using a Shim-pack GIST PFPP Kyoto, Japan (2.1 mm I.D.×150 mm, 3.0 mm) column and a gradient system with the mobile phase consisting of solvent water and acetonitrile with 0.1% of formic acid in each solvent, at a flow rate of 0.25 mL/min, and an injection volume of 3 µL. The gradient program used 0–2 min 100% of A, 5 min 75% of A, 11 min 65% of A, 16 min 50%, 19 min 5%, 30 min 100% of B for 2 min, and hold for 4 min. The conditions of mass spectroscopy were in ESI positive and negative modes.

Analysis of Fatty acids

Analysis of the chemical composition of fatty acids involves transesterification of glycerides into volatile methyl esters. The hexanoic extract of the plant was prepared using the Soxhlet extraction. Then the extract was concentrated using rotavapor. 0.5 ml of methanolic potassium hydroxide with a normality of two and 10 ml of methanol was introduced to the extract. The resultant mixture is then subjected to reflux heating for 15 minutes until achieving a clear solution. Following cooling, 1 ml of heptane is incorporated into the reaction mixture. The phase containing the methyl esters in heptane is transferred to a test tube, where a solution

of sodium carbonate is introduced. This sodium carbonate solution neutralizes any free acids present, forming sodium salts and releasing carbon dioxide in the process. These compounds are analyzed using gas chromatography (GC). The Shimadzu GCMS-TQ8040 chromatograph, coupled with a mass spectrometer (GC-MS) system, is employed. The injector maintained at 240°C, with helium serving as the carrier gas. Analysis is conducted through temperature programming, starting at 140°C and ramping up to 200°C at a rate of 10°C per minute, followed by an isothermal hold at 200°C for 60 minutes. A Restek Rtx-5MS fused-silica column, with dimensions of 30 meters in length, 0.25 mm internal diameter, and 0.25 µm film thickness, is utilized. The mass spectrometer operates in full scan mode, and compound identification is achieved using the NIST 2017 database.

Total protein contents

Proteins were determined according to the Bradford assay. Plant extract (0.1 mL supernatant) was added to 0.1 mL water and 2 mL Bradford reagent in a test tube. The mixture was homogenized, and the absorbance recorded at 595 nm after 1 min. BSA was used as a standard, and proteins content was given as mg equivalent BSA/g Extract.

Antioxidant activities in vitro

DPPH free radical scavenging activity

The free radical scavenging DPPH test was carried out according to the method carried out by [1]. A variation in concentration for each plant extracts was added to a 0.2 mM solution of DPPH in methanol and the reaction mixture was stirred. The amount of DPPH remaining was determined at 517 nm and calculate using the following equation:

$$\% \text{ DPPH} = [\text{A control} - \text{A sample/A control}] \times 100]$$

The test was carried out three times and the average of the percentage reduction of the DPPH radical and the concentration responsible for 50% of its inhibition (IC_{50}) were determined.

Ferric reducing antioxidant power

The FRAP method is based on the ability to modify oxidation transition metals, it is measured by the Fe (III) -TPTZ complex [2,4,6-Tris (2-pyridyl) -s-triazine] by the determination of ferric ions reducing antioxidant power (FRAP) or the method has been modified for the microplate screening system [2]. The oxidation capacity of the condition of transition metals was measured using the based on the reduction of the K3 complex [Fe $(CN)_6$]. 30 µL of each extract has been mixed with 100 µL distilled water, 45 µL distilled water 1M HCl, 45 µL of 1% (w/v) K3 [Fe $(CN)_6$], 15 µL of 1% (w/v) 0.2% SDS and 15 µL (w/v) $FeCl_3$ Solution. The reaction mixture was incubated for 20 minutes at 50°C, followed by an absorbance measurement at 700 nm. Antioxidant activity was determined to be equivalent to mM $FeSO_4$/mg extract [1].

References

1. Ghareeb, M.A.; Mohamed, T.; Saad, A.M.; Refahy, L.A.-G.; Sobeh, M.; Wink, M. HPLC-DAD-ESI-MS/MS Analysis of Fruits from Firmiana Simplex (L.) and Evaluation of Their Antioxidant and Antigenotoxic Properties. *Journal of Pharmacy and Pharmacology* **2018**, *70*, 133–142.
2. Kulichová, K.; Sokol, J.; Nemeček, P.; Maliarová, M.; Maliar, T.; Havrlentová, M.; Kraic, J. Phenolic Compounds and Biological Activities of Rye (Secale Cereale L.) Grains. *Open Chemistry* **2019**, *17*, 988–999.
